# Regional analysis of APOE polymorphism in Alzheimer’s disease in Spain

**DOI:** 10.1038/s41598-025-98323-2

**Published:** 2025-04-28

**Authors:** Ignacio Pardo-Pérez, Leticia Sánchez-Valdeón, Ana García-Gallego, Brisamar Estébanez, Inés Casado-Verdejo, Jesús Antonio Fernández-Fernández, Carlos Méndez-Martínez, Laura Bello-Corral

**Affiliations:** 1 University Hospital of León, 24071 León, Spain; 2 Department of Nursing and Physiotherapy, University of León, 24071 León, Spain; 3 Health Research Nursing Group (GREIS), University of León, Campus Vegazana, 24071 León, Spain; 4 Department of Economics and Statistics, University of León, 24071 León, Spain; 5 Institute of Biomedicine (IBIOMED), University of León, 24071 León, Spain; 6 Department of Nursing and Physiotherapy, University of León, 24401 Ponferrada, Spain

**Keywords:** Alzheimer’s Disease, *APOE* polymorphism, Genetic, Geographical distribution, Life quality, Genetics, Neuroscience, Biomarkers, Neurology

## Abstract

Alzheimer’s disease (AD) is a progressive neurodegenerative disorder with significant cognitive and functional impacts. Genetic factors, particularly the *APOE* gene and its allelic variants (ε2, ε3, ε4), play a critical role in AD susceptibility. This study analyzed the allelic frequency and distribution of *APOE* polymorphisms in three provinces of Castilla y León (León, Soria, Salamanca), Spain, to explore their potential relationship with AD risk. Genotypes were determined using polymerase chain reactions, and statistical analyses revealed significant regional variations. The ε3/ε3 genotype was the most prevalent overall, while the ε3/ε4 genotype predominated in specific areas like Ponferrada. The absence of homozygous ε4 individuals in Soria contrasts sharply with higher frequencies in Salamanca. These differences suggest historical and migratory influences on genetic variability. Identifying regional genetic patterns enhances our understanding of AD risk and supports the development of targeted preventive strategies. Early detection of high-risk alleles could improve patient outcomes, reduce healthcare burdens and inform public health policies.

## Introduction

First described by Alois Alzheimer in 1907, Alzheimer’s disease (AD) is a progressive and irreversible neurodegenerative condition that mainly affects cognitive functions, memory and behaviour, gradually rendering individuals more vulnerable and dependent. This progression places a continuous burden on both family members and caregivers^1^.

According to the World Health Organization (WHO), an estimated 55.2 million people worldwide were affected by AD and other dementias in 2019. This number is expected to rise to 75 million by 2030 and 132 million by 2050. Although the risk of dementia may decrease as social conditions improve, due to coordinated efforts to reduce risk factors and promote protective measures, it is important to note that the European region currently has the second highest number of dementia cases, with a total of 14.1 million^2^.

Research conducted in Spain estimates the prevalence of AD to be approximately 0.05% among individuals aged 40–64 years; 1.07% in the 65–69 age range; 3.4% for those aged 70–74 years; 6.9% in the 75–79 age group; 12.1% for individuals aged 80–84 years; 20.1% in the 85–89 age group; and 39.2% among those over 90 years of age^3^.

When analyzing the brain of a patient with AD from a pathological anatomy perspective, generalized, bilateral and symmetrical atrophy is observed, with prominent involvement of the hippocampus, amygdala and fronto-temporo-parietal areas, which are recognized as association areas^4^.

At the microscopic level, AD is characterized by two types of abnormal structures. Senile plaques primarily consist of deposits of β-amyloid (Aβ). A 36–43 amino acid peptide precursor of amyloid precursor protein (APP), which abnormally folds into extracellular nest-like lattices. The Aβ42 variant is strongly associated with early amyloid deposition in the extracellular neocortex, which subsequently spreads to subcortical structures such as the hippocampus and entorhinal cortex, impairing cognitive function^5,6^. Neurofibrillary tangles (NFBs) are composed of Tau protein, which becomes abnormally folded and hyperphosphorylated. These tangles consist of long unbranched neurofilaments located within the cytoplasm of neurons. The distribution of NFBs follows the Braak and Braak Scheme, with the highest concentration found in the limbic system, which affects physiological and emotional responses^5,7,8^.

Recent research indicates that in 95% of AD cases, classified as late-onset AD (LOAD), can be attributed to two key categories of risk factors: modifiable and non-modifiable^9^ . Modifiable risk factors include conditions such as type 2 diabetes *mellitus*, high blood pressure and obesity, while non-modifiable risk factors encompass age, gender, ethnic background and the presence of the apolipoprotein epsilon (*APOE*)^10,11^.

Over the past decade, significant research has uncovered various biomarkers, such as Tau protein, detectable in urine, blood, cerebrospinal fluid, tears and saliva, that aid in the early diagnosis of AD. Among these, the *APOE* gene has been prominently identified in the scientific literature as a crucial possible biomarker and a key indicator of the disease^12–14^.

The *APOE* gene is located within a 45kb gene cluster on chromosome 19, at the q13.2-q13.3 locus^15^. It encodes *APOE*, a 34kDa glycoprotein composed of 299 amino acids, with if primarily produced by astrocytes under physiological conditions. Its essential function is to protect neurons from oxidative stress^16^.

The *APOE* gene locus is polymorphic, presenting three possible isoforms: *APOE*2, *APOE*3 and *APOE*4. These correspond to three alleles of the gene, ε2, ε3 and ε4, which result in six possible genotypes that are inherited in a codominance manner^11^. Although the isoforms differ only by the position of two amino acid residues, their structural conformations vary significantly^17^.

**Fig. 1:**
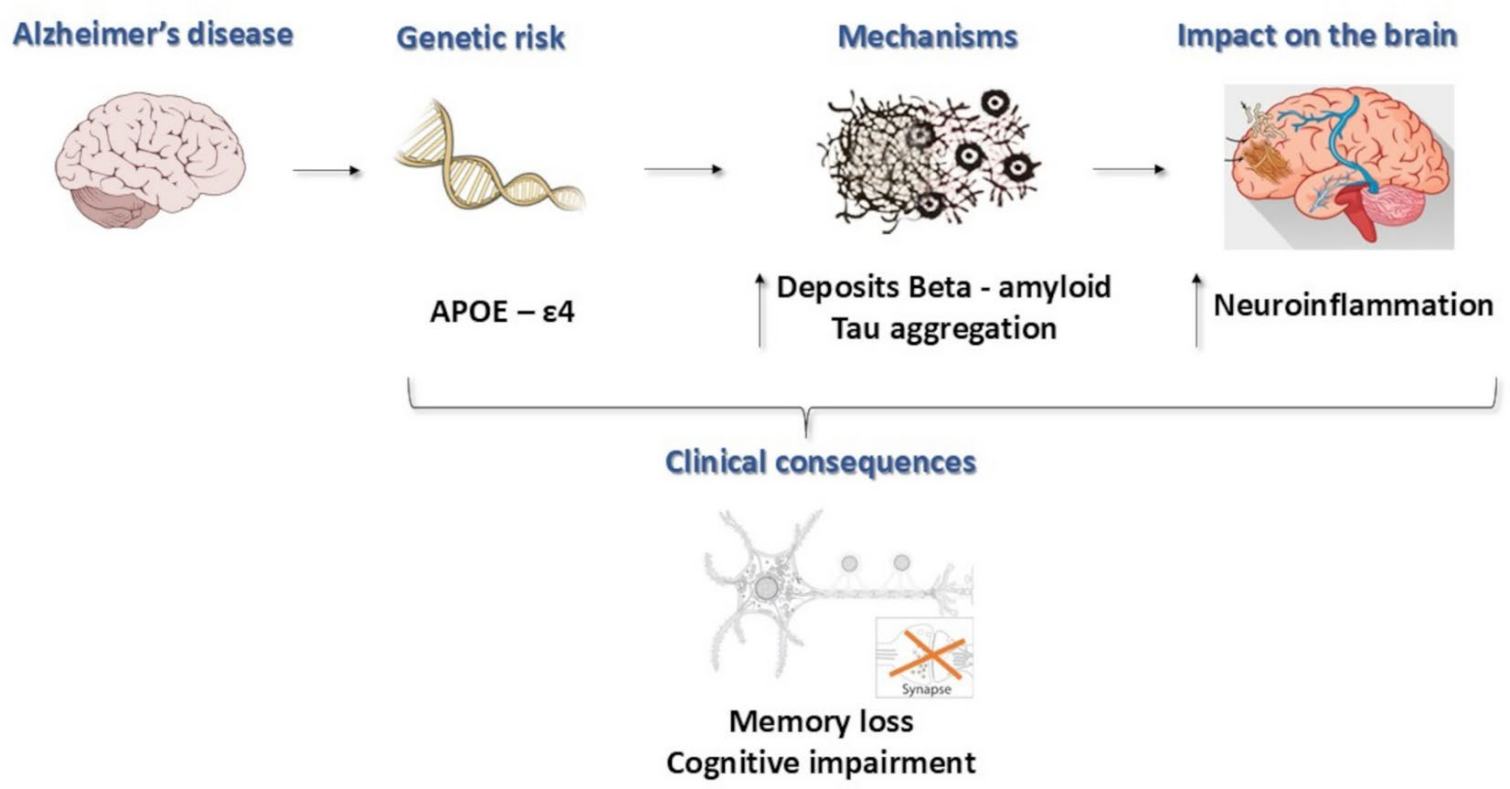
Effects of *APOE*4 in Alzheimer’s Disease. The presence of the *APOE*4 isoform promotes the formation of β-amyloid, which in turn increases the formation of senile plaques and the aggregation of Tau protein. This leads to heightened neuroinflammation, resulting in clinical consequences such as neuronal loss and cognitive decline. Abbreviation: *APOE*: Apolipoprotein E;

*APOE*4 is associated with a heightened risk of AD (Fig. 1), with heterozygous carriers having a 3- to fourfold increased risk and homozygous carriers facing an 8- to 12-fold increase. In contrast, *APOE*2 is considered protective against AD, linked to reduced hippocampal atrophy and lower concentrations of Tau protein ^17^.

**Fig. 2:**
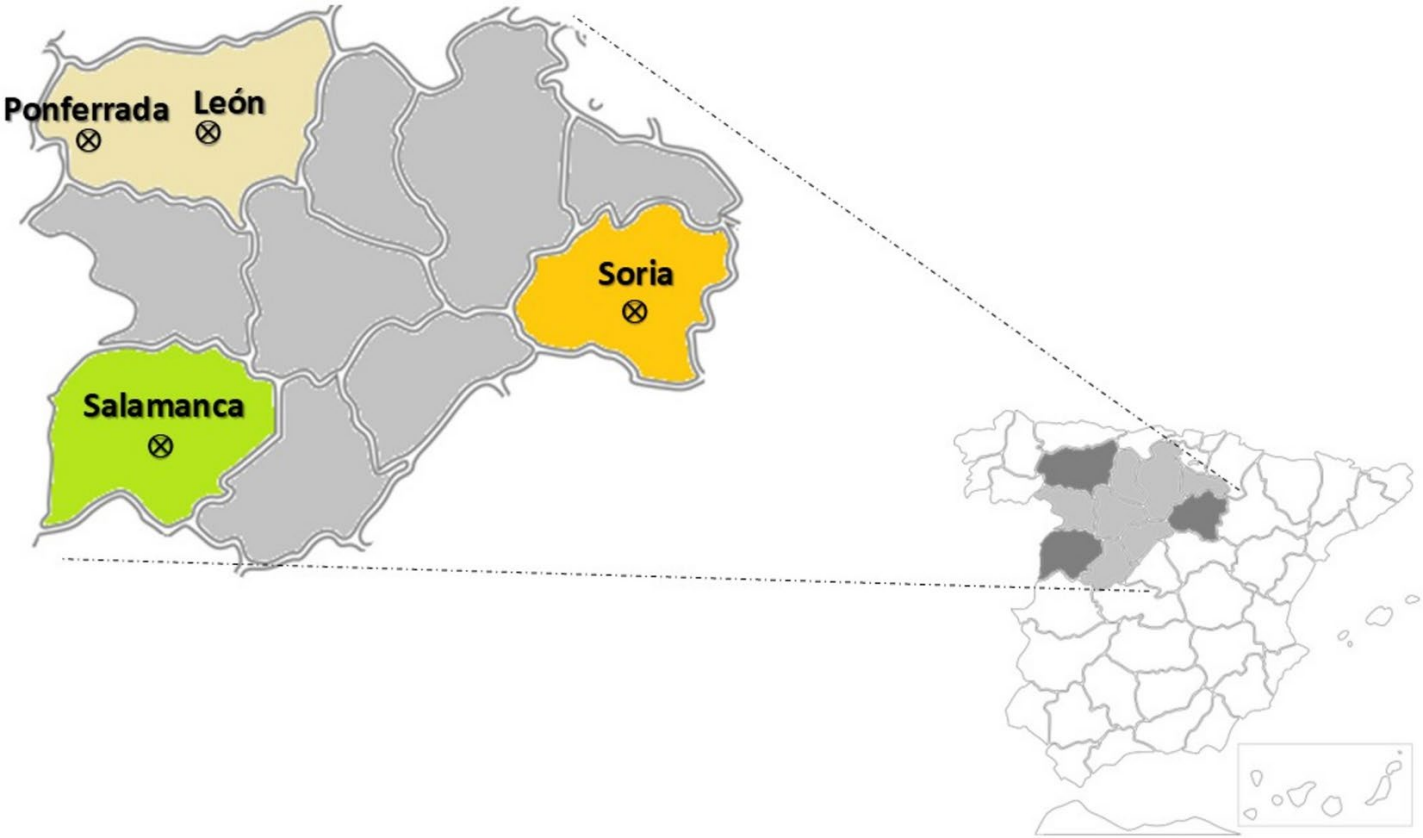
Map showing the Iberian Peninsula with the location of the provinces of León, Soria, and Salamanca in Castilla y León.

The geographical distribution of *APOE* gene in Spain reflects a complex phenomenon shaped by a long and intricate history^18,19^. Castilla y León is one of the autonomous communities with the oldest population in Spain^20^, making it a relevant setting for studying AD and its genetic risk factors. Despite the significance of genetic influences on AD, studies on the *APOE* polymorphism in Spain remain scarce. Due to its geographical location at a crossroads, Castilla y León has received significant genetic contributions from Northern Europe, the Mediterranean area, and Northern Africa. These historical influences may have shaped the distribution of *APOE* alleles in this region, highlighting the importance of analyzing their impact on AD risk. Understanding the *APOE* gene polymorphism in this population is crucial for assessing its potential role in AD susceptibility. Given the demographic characteristics of Castilla y León and its genetic background, examining the distribution of *APOE* alleles in the provinces of León, Soria and Salamanca (Fig. 2) may provide valuable insights for developing targeted prevention and care strategies. Characterizing the *APOE* gene polymorphism in this population will not only help identify individuals at higher genetic risk but also support the development of personalized prevention and care approaches in the region’s daycare centers. Therefore, this study aims to expand the current knowledge regarding the presence, allelic frequency and genetic distribution of the *APOE* gene in the provinces of León, Soria and Salamanca, while comparing the data obtained with results from similar studies conducted in other regions of Spain and Europe.

## Methods

### Study design

An analytical observational case–control study carried out in the "Mensajeros de la Paz" Residences and Asociaciones de Enfermos de Alzheimer (AFA) in Castilla y León, following the guidelines of the Strengthening the Reporting of Observational Studies in Epidemiology (STROBE) statement were followed. This study setting was selected due to their broad patient population, providing a suitable sample for the study. Additionally, Castilla y León’s historical position as a geographical crossroads has contributed to a diverse genetic background, reinforcing the relevance of analyzing the distribution of *APOE* polymorphisms in these regions.

### Ethical considerations

Prior to the initiation of this study, approval was obtained from the Ethics Committee of the University of León (code ETICA-ULE-021–2022), ensuring full compliance with the legal requirements set forth by Law 31/1995 on the Prevention of Occupational Hazards and Law 14/2007 on Biomedical Research concerning the use of human biological samples. The research adhered to the ethical standards outlined in the Declaration of Helsinki, including its subsequent revisions, and was registered in ClinicalTrials.gov under code NCT06275243.

### Study cohort

In the case group, a total of 260 people diagnosed with AD were included, sourced from the AFA of Castilla y León, as well as users of the "Mensajeros de la Paz" Residences located in Mansilla de las Mulas (León) and in La Bañeza (León).

In the control group, a total of 251 people without a diagnosis of AD were included. These participants were recruited in the AFA of León and Ponferrada, as well as in the University of Experience in León and Ponferrada and in the Maintenance Gymnastics classes offered by the City Council of León.

### Inclusion and exclusion criteria

Recruitment inclusion criteria were: (i) Age 60–90 years; (ii) Case group with diagnosis of AD; (iii) Control group without diagnosis of AD; (iv) Voluntarily participation and free of charge. The exclusion criteria were: (i) Participants who, during sampling, presented behavioural and oral alterations or other conditions that impeded the process.

### Sample collection and processing

The Canvax® Buccal swab collection & stabilization kit was used to collect buccal cheek cells using a swab. The sample was suspended in an Eppendorf tube with stabilisation fluid and centrifuged at 13,000 RPM for 5 min. Then, 10–20 µl of residual liquid was left for preservation.

Samples were processed with the Canvax® Buccal swab genomic DNA extraction kit to purify the DNA. 3 ml of proteinase K and 200 µl of buffer were added to the sample, homogenised and incubated at 55°C for 1 h. Afterwards, S3 buffer was added, vortexed and centrifuged. The resulting liquid was mixed with isopropanol and buffer S4, centrifuged and the supernatant removed. The supernatant was washed with 70% ethanol and centrifuged again. Finally, the supernatant was removed and allowed to dry.

To amplify *APOE*2, *APOE*3 and *APOE*4 isoforms by polymerase chain reactions (PCR), the sequence-specific primer method described by Pantelidis et al.^21^ was followed. Three PCR reactions were performed with different combinations of E2, E3 and E4 primers and control primers. The results were analysed by 2% agarose gel electrophoresis with ultraviolet visualisation, with the presence of a 173 bp band being specific and a 785 bp band being control.

### Statistical analysis

A descriptive statistical analysis was performed by tabulating the data according to their distribution by absolute and relative frequencies, and they were represented graphically to obtain visual conclusions.

In addition, an inferential study was carried out to test hypotheses about differences between individuals with AD and the control group. To assess significant differences in quantitative variables, the t-test for independent samples was used, assuming normal populations and verifying normality with Kolmogorov–Smirnov and Shapiro–Wilk tests. In the case of several groups, the alternative ANOVA method was used, applying the Sheffé’s test for multiple comparisons. The chi-square test or exact test was used for qualitative variables.

Statistical significance was considered at a p-value of less than 0.05.

All analyses were performed with IBM SPSS Statistics for Windows, version 26.0 (Armonk. NY: IBM Corp.).

## Results

The study population comprised 511 individuals, with 260 diagnosed with AD and the remaining 251 identified as healthy controls. The 260 participants with AD were recruited from various AFA located in Soria, Salamanca, León and Ponferrada, as well as from the "Mensajeros de la Paz" Residences in Mansilla de las Mulas (León) and La Bañeza (León).

### Socio-demographic data

After obtaining the population sample and conducting personal interviews with the 260 individuals in the case group and 251 in the control group about their socio-demographic characteristics, the following results were obtained and are shown in Table 1.

**Table 1:** Socio-demographic data by group.

Characteristics		AD case group		Control group		p-value
		*Frequency*	***%***	*Frequency*	***%***	
**Sex**	***Men***	62	23,85	41	16,33	0,0343*
	***Women***	198	76,15	210	83,67	
**Civil status**	***Single***	12	5,02	22	8,91	< 0,0001***
	***Married***	100	41,84	157	63,56	
	***Widowed***	125	52,30	50	20,24	
	***Divorced***	1	0,42	13	5,26	
	***Separated***	1	0,42	5	2,02	
	***Not available***	21	–	4	–	
**Educational level**	***Without studies***	7	3,68	0	0	< 0,0001***
	***Primary***	142	74,74	54	22,98	
	***Secondary***	16	8,42	98	41,70	
	***Higher***	25	13,16	83	35,32	
	***Not available***	70	–	16	–	
**Residential area**	***Rural***	65	27,58	11	4,44	< 0,0001***
	***Urban***	175	72,92	237	95,56	
	***Not available***	20	–	3	–	
**Family history of AD**	***Yes***	88	45,60	65	26,75	0,0002***
	***No***	101	52,30	174	71,60	
	***Unknown***	4	2,10	4	1,65	
	***Not available***	8	–	8	–	

Note: z test for the variables of sex and municipality of residence; chi-2 test for the variables of marital status, educational level and history of. Abbreviation: AD: Alzheimer’s Disease.

In both groups, the percentage of women is higher than that of men, being more notable in the control group with 83.67% of women. The AD case group has a higher proportion of widowers (52.30%) compared to the control group (20.24%), while the control group has a higher proportion of married people (63.56%) compared to the case group (41.84%).

In the case group, 74.74% have primary education, while in the control group, the highest level is secondary education with 41.70%. The majority of both groups reside in urban areas, being higher in the control group (95.56%) compared to the case group (72.92%).

An 45.60% of the case group had a family history of AD, in contrast to 26.75% of the control group.

### Distribution of APOE genotypes by provinces in Castilla y León

In this section, we analyze how genotypes are distributed across the provinces of Castilla y León. To do so, we consider the sample of AD cases, as the focus is on studying the provincial distribution of genotypes among patients with AD.

First, we describe the sociodemographic characteristics of this sample of patients, which are presented in Table 2, analyzing potential differences by genotype.

**Table 2:** Distribution of *APOE* genotypes by demographic characteristics.

	Sex		Age	Educational level				Civil status					Residential area		Family history of AD		
GENO TYPE	Men (%)	Women (%)	Mean ± SD	No studies	Primary	Secondary	Higher	Single	Married	Widowed	Divorced	Separated	Rural	Urban	Yes	No	**Unknown**
ε2/ε2																	
ε2/ε3	3	11	84.38 ± 7.68	2	7	0	2	0	4	8	0	0	1	12	7	5	0
ε2/ε4	1	2	89.00 ± 1.00	0	3	0	0	0	2	1	0	0	0	3	0	2	0
ε3/ε3	32	107	85.53 ± 7.58	2	79	7	9	6	49	74	0	1	42	88	41	54	3
ε3/ε4	24	65	82.10 ± 7.16	3	46	8	11	5	39	35	1	0	18	62	34	35	1
ε4/ε4	1	11	77.91 ± 4.97	0	6	1	3	1	5	5	0	0	2	9	5	5	0
p-value	**0.646**		< 0.0001***	**0.213**				**0.635**					**0.789**		**0.826**		

Note: exact test for the variables of sex, educational level, residential area, marital status, and history of AD. ANOVA test for the variable of age. Abbreviation: AD: Alzheimer’s Disease.

As shown, no significant differences are observed by genotype in terms of sociodemographic variables, except for age, where significant differences are found at a level below 0.001. Applying Scheffé’s test, the groups with the greatest differences are the ε3/ε3, ε3/ε4 and ε4/ε4 genotypes.

**Fig. 3:**
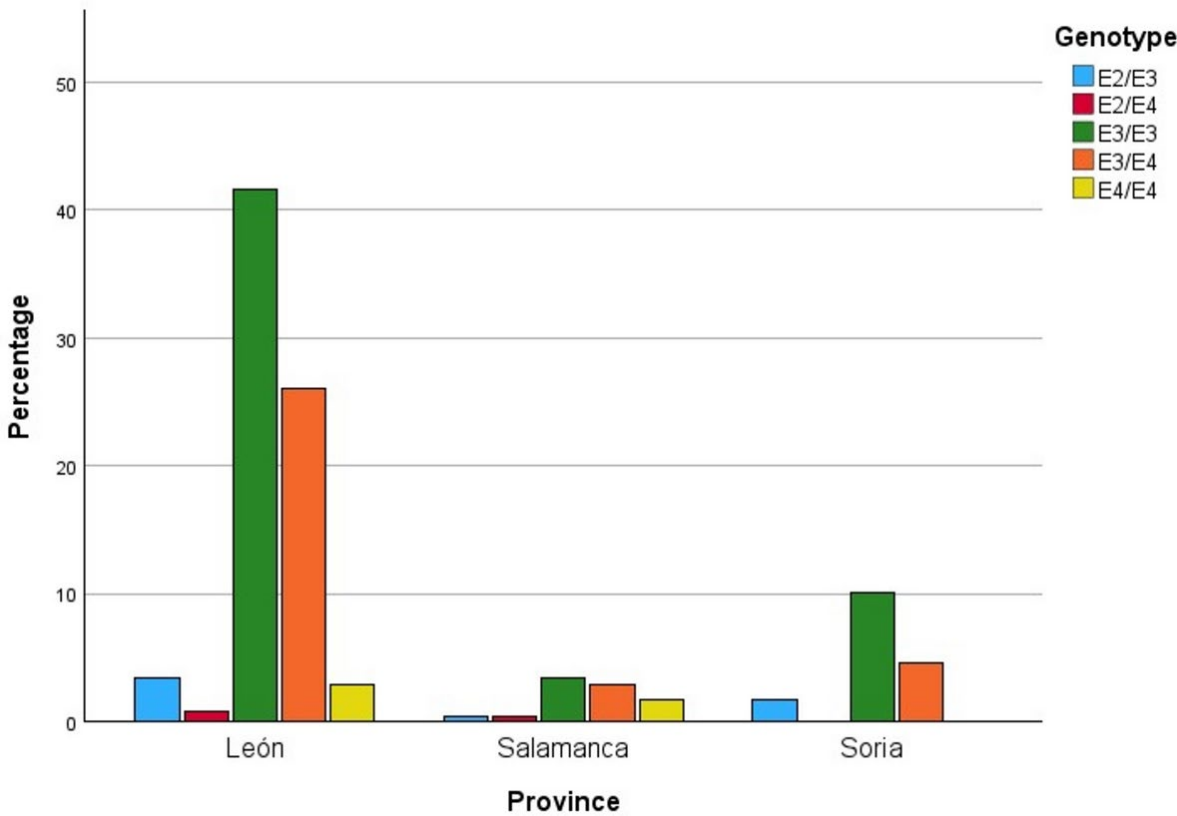
Distribution of genotypes among individuals with AD by province of residence.

Information on the distribution of genotypes by geographical area is shown in Fig. 3, as well as in Table 3.

**Table 3:** Distribution of genotypes of AD cases by geographical area.

	León						Salamanca		Soria	
GENOTYPE	*Frecuency*			*%*						
	León	Ponferrada	Province	León	Ponferrada	Total	*Frecuency*	*%*	*Frecuency*	*%*
ε2/ε2	*0*	*0*	0	*0*	*0*	0	0	0	0	0
ε2/ε3	*7*	*1*	8	*5,22*	*2,27*	4,50	1	4,76	4	10,26
ε2/ε4	*1*	*1*	2	*0,75*	*2,27*	1,10	1	4,76	0	0
ε3/ε3	*80*	*19*	99	*59,70*	*43,18*	55,60	8	38,10	24	61,54
ε3/ε4	*42*	*20*	62	*31,34*	*45,45*	34,80	7	33,33	11	28,21
ε4/ε4	*4*	*3*	7	*2,99*	*6,82*	3,90	4	19,05	0	0
Total	*134*	*44*	178	*100*	*100*	100	21	100	39	100

As can be seen, the most frequent genotype in the three provinces is the homozygous ε3, especially in Soria, with a percentage of over 60%. This is followed by the ε3/ε4 genotype, which in the case of Salamanca presents a percentage of cases quite close to the previous genotype (38.10% compared to 33.33%). As for León, the situation is similar to the other two provinces, with 55.60% of individuals carrying the ε3 homozygote and 34.80% carrying the ε3/ε4 genotype.

**Fig. 4:**
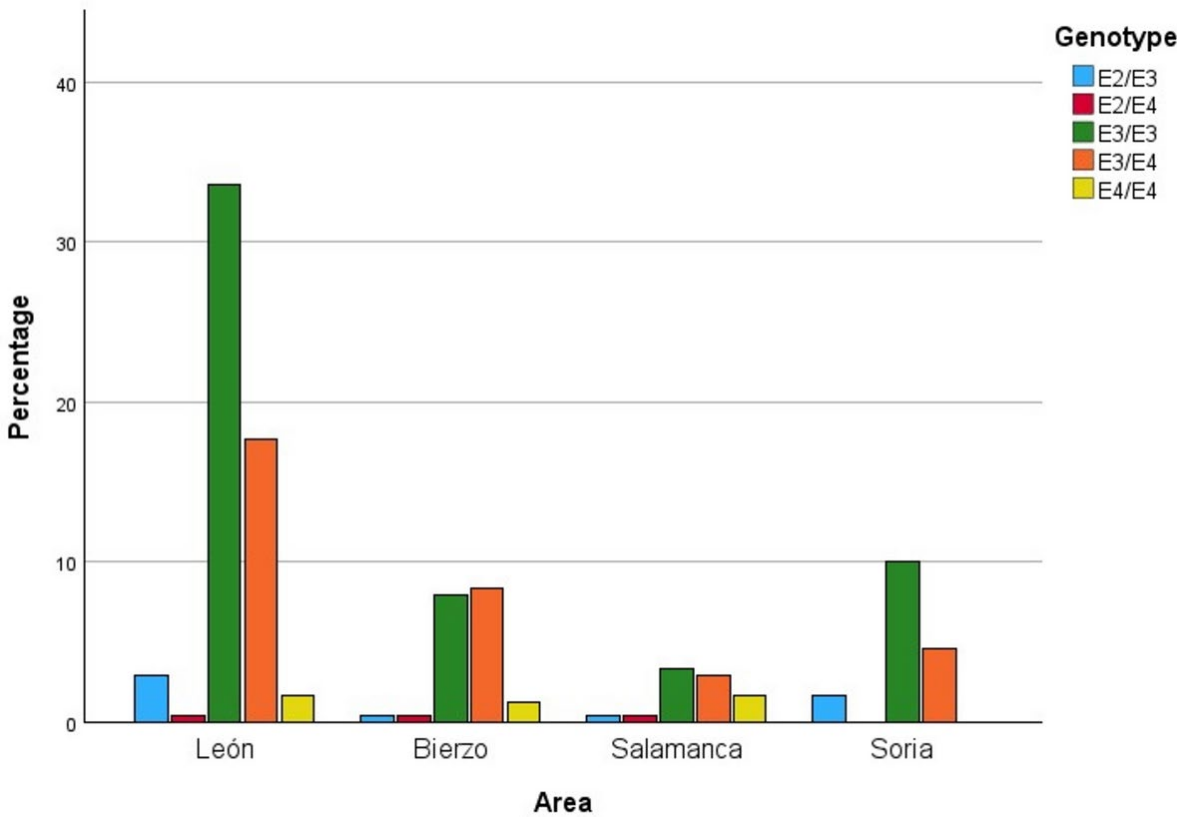
Distribution of genotypes among individuals with AD by area.

However, when considering the area of León and Ponferrada separately, some differences are observed. The area of León shows the same trend as described above, with percentages similar to those of Soria (59.70% and 31.34% for each genotype, respectively). In Ponferrada, on the contrary, the most numerous genotypes are ε3/ε4, followed by ε3/ε3, although the percentages are similar (45.45% and 43.18%, respectively) as shown in Fig. 4. To confirm there are no differences between these two areas, a z-test for independent proportions was applied. In the case of genotype ε3/ ε4, a p-value of 0.7083 was obtained, whereas for the genotype ε4/ ε4, the p-value was 0.8366. Therefore, the percentage of individuals with these two genotypes can be considered as similar.

Finally, the homozygous ε4 genotype is not present in the sample of individuals from Soria. In the province of León, it is found in 3.90% of cases, although the percentage is higher in the Ponferrada area than in León (6.82% compared to 2.99%). In Salamanca it is 19.05%.

These results are consistent with those obtained by González et al.^22^, who considered a sample of patients from the Castilla y León region. Applying a z-test for the difference in proportions between the percentages of AD patients in our study and those in González et al.^22^ for each genotype, no significant differences were observed, as the p-value was greater than 0.05 in all cases.

Another study that analyzed the distribution of genotypes by province was conducted by Reales et al.^23^, focusing on the Andalusia region, specifically the provinces of Granada and Huelva. Applying the same test, significant differences were observed between the two studies. Specifically, in both Granada and Huelva, there is a significantly lower percentage of individuals with the ε3/ε4 genotype (p-value < 0.001***), while the percentage of cases with the ε3/ε3 genotype is significantly higher (p-value < 0.01** and p-value < 0.001***, respectively). Additionally, in the case of Granada, there is a significantly lower percentage of individuals with the ε4/ε4 genotype compared to our study (p-value < 0.05*).

## Discussion

The aim of this study was to analyze the allelic and genotypic frequencies and genetic distribution of the *APOE* gene in the provinces of León, Soria, and Salamanca, with a focus on understanding its implications for the prevention and management of AD. To provide a broader context, the findings were compared with similar studies conducted in other regions of Spain and Europe. Given the growing relevance of *APOE* in biomedical research, particularly in neurodegenerative diseases like AD, a genetic analysis was performed on individuals from the AFA associations in Soria, Salamanca, León, and Ponferrada, as well as from ‘Mensajeros de la Paz’ Residences in Mansilla de las Mulas and La Bañeza (León).

A total of 511 individuals were evaluated, of whom 260 had been diagnosed with AD and 251 were healthy. In both groups, the proportion of women was higher, reflecting the general trend of longer life expectancy in women and their greater susceptibility to AD^24^.

The higher proportion of widowed and the lower proportion of married people in the case group, as well as the reverse situation in the control group, indicate that marital status has a significant impact on the incidence of AD. Widowhood is associated with an increased risk of social isolation and emotional stress, factors that may contribute to cognitive decline. Conversely, being married may offer a protective effect, due to increased social support and continuous cognitive stimulation^25,26^.

The higher prevalence of lower educational levels in the case group suggests a correlation between lower educational level and higher risk of AD. The results obtained support the cognitive reserve hypothesis, which posits that higher education provides a greater ability to compensate for pathological changes in the brain^27,28^. Related to this is the higher proportion of AD cases residing in rural settings.

Analysis of *APOE* gene genotype frequencies in the provinces of León (including León and Ponferrada), Salamanca and Soria provides valuable information on the public health of Castilla y León in terms of the distribution of ε2, ε3 and ε4 allelic variables.

The most prevalent genotype in all populations was ε3/ε3, corresponding to 55.60% in León, 38.10% in Salamanca and 61.54% in Soria. The results show the expected correlation with what has been studied so far about the expression of the *APOE* locus, since the ε3 allele is considered to be the most prevalent^29^. As expected, the ε2/ ε2 genotype was found at the lowest percentage in all populations (0%), coinciding with the results obtained for Castilla y León by González et al.^22^ in 2020, and being similar to those included in the study by Reales^23^ for the populations of Huelva and Granada (0.66% and 0.59%, respectively). These results contradict the pre-established notion of a positive clinal distribution of the ε2 allelic variant towards southern Europe^30^.

Regarding the distribution of the ε4/ε4 genotype, the values observed in Leon (3.90%) and especially in Soria (0%), reflect the latitudinal trend in the European continent, where there is a positive gradient from south to north ^30^. It is worth noting that the 19.05% of the sample studied in Salamanca for this genetic variant represents one of the highest percentages reported to date in Spain.

Our findings regarding the distribution of the *APOE* alleles align with previous studies conducted in Spain and other European populations. For instance, our results confirm that the ε3 allele is the most prevalent in all studied provinces, consistent with findings from González et al.^22^ in Castilla y León. Additionally, the frequency of the ε4 allele follows the expected latitudinal trend observed in Europe, where its prevalence increases from south to north, as reported by Corbo & Scacchi^30^. However, our study also highlights some unexpected variations, such as the notably high ε4/ε4 genotype frequency in Salamanca (19.05%), which differs from previous reports in other Spanish regions, such as Barcelona, where Haddy et al.^31^ reported an ε4 allele frequency of 11%, or Galicia, where Garcés et al.^32^ defined this frequency as 8.30%. This indicates that the distribution of *APOE* gene polymorphism in the Iberian Peninsula is less predictable from a geographical perspective, as no clear meridional or latitudinal gradient is discernible.

Spain’s strategic location at the crossroads between Europe and Africa has facilitated significant interactions among diverse ethnic and cultural groups. As a result, each population that settled in Spain throughout various historical periods has left both a historical and genetic legacy. Prior research has suggested that the risk associated with the ApoE4 allele may be attenuated in individuals of African ancestry compared to those of European descent. Specifically, Rajabli et al.^33^ identified that African genetic ancestry at certain loci (17p13.2) may reduce the risk of AD in African American populations, though the overall proportion of African ancestry alone does not fully explain differences in AD prevalence. However, in our study, we did not assess the genetic ancestry of participants, and therefore, we cannot determine whether the African genetic legacy in different Spanish regions influences the distribution of *APOE*4 or its association with AD risk. Future studies incorporating ancestry-informative markers could provide valuable insights into how genetic background modulates the effects of *APOE*4 on AD risk across different regions.

The ε2/ε4 genotype is particularly rare due to the low frequency of the ε2 and ε4 alleles individually and the low probability of both being present in the same individual. This explains the percentages of 0% in Soria, 0.75% in León, 2.27% in Ponferrada and 4.76% in Salamanca.

The alleles of the* APOE* gene (ε2, ε3 and ε4) have different evolutionary origins. The ε4 allele is the most ancestral, present in the earliest human populations. With dispersal and adaptation to new environments, the ε3 and ε2 alleles emerged. Currently, the ε3 allele is the most common and provides an adaptive advantage in lipid metabolism. The ε2 allele, being more modern and less common, is associated with life extension and increased responsiveness to dietary fat^34^.

The frequency of the ε4 allele is determined by the percentages of the ε4/ ε4 and ε3/ ε4 genotypes. The difference between the León and Ponferrada areas for these two genotypes is particularly relevant: 2.99% and 6.82% respectively for ε4/ε4, as well as 31.34% and 45.45% respectively for ε3/ ε4. This may suggest a persistence of ancestral characteristics in this population due to factors such as geographical isolation and limited migratory flow from the Ponferrada area.

The low frequency of the ε2 allele supports the premise of being the most modern. Therefore, it is possible that the adaptive advantages it offers are not yet available to all populations.

The higher frequency of the ε4 allele in a province could be associated with an increased risk of developing AD in its population, as this allele is the main known genetic risk factor for the disease. Several studies have shown that carriers of the ε4 allele have a higher risk of developing Alzheimer’s at earlier ages compared to non-carriers^35,36^. While genetics play a crucial role, other modifiable and environmental factors also influence the development of AD in populations with a higher prevalence of the ε4 allele. Cardiovascular risk factors, such as high blood pressure, diabetes mellitus and hypercholesterolemia, as well as unhealthy dietary patterns, have been associated with both *APOE*4 and an increased risk of AD^37,38^. However, research also suggests that lifestyle interventions can mitigate this genetic risk. Adherence to a healthy lifestyle, including regular physical activity, a balanced diet, such as the Mediterranean diet, maintaining social engagement, participating in cognitively stimulating activities, and avoiding smoking and excessive alcohol consumption, has been associated with a slower rate of memory decline, even among *APOE*-ε4 carriers^39^.

Given the increased risk of AD associated with the *APOE*-ε4 allele, particularly in populations with a higher prevalence, public health strategies should emphasize early screening, lifestyle modifications, and preventive interventions. Evidence suggests that adherence to a structured lifestyle program—including a whole-food, minimally processed plant-based diet, regular aerobic and strength-training exercise, stress management techniques (e.g., meditation, yoga, and relaxation exercises), cognitive stimulation, and social support—can contribute to cognitive health and mitigate genetic risk factors. Additionally, targeted nutritional supplementation (e.g., omega-3 fatty acids, curcumin, vitamin B_12_, and magnesium) has been explored for its potential neuroprotective effects^40^. Implementing these interventions through public health initiatives could be particularly beneficial for at-risk populations, promoting brain health and reducing the burden of AD.

## Conclusions

The results show significant differences in genotypic frequencies, suggesting genetic variability influenced by historical and migratory factors specific to each region. These findings reflect the persistence of ancestral characteristics in Ponferrada and Salamanca. Furthermore, the low frequency of the ε2 allele suggests reduced genetic protection against AD in the studied population, supporting its classification as a more modern variant.

Additionally, differences in AD prevalence between rural and urban areas underscore the need to enhance access to specialized health services and education about the disease in rural regions. The distribution patterns of *APOE* gene alleles in Castilla y León are consistent with trends observed in other European populations, facilitating comparisons with genetic studies across Europe.

Early identification of risk alleles enables the implementation of preventive and therapeutic strategies for AD. This, in turn, supports the development of effective prevention programs, improves patient’s quality of life and alleviates the burden on their families and the healthcare system.

## Data Availability

The original contributions presented in the study are included in the article, further inquiries can be directed to the corresponding author.
